# Aids to Nursing

**Published:** 1888-03-17

**Authors:** M. M. Basil

**Affiliations:** Author of "The Commoner Accidents to Life and Limb"


### AIDS TO NURSING.

By M. M. BASIL, M.A., M.B., C.M., Author of "The Commoner Accidents to Life and Limb."

*(Continued from p. 396.)*

### NOISE.

THE freedom of the sick-room from all unnecessary noises and disturbances is a matter of considerable importance. In many diseases, especially such as interfere primarily with the nervous system, or cause exhaustion with irritability, there is an over-sensitiveness of the special organs, and particularly of the organ of hearing. Extreme caution is, therefore, necessary to maintain absolute quiet. All sounds, however, are not equally injurious. The patient can often bear certain sounds well enough, while others are acutely painful. Noises that are unnecessary, or such as create an expectation in the mind of the patient and then disappoint him, are those which do most harm. Thus, for example, people who have lived in towns can bear the noise of the traffic well enough ; but rough walking, clattering of plates, ringing of church bells, or talking in the next room, harm them greatly. Some of the sounds which are very annoying to patients are such as the crackling of starched clothing, the fidget of silk, rattling of keys, jingling of cups and spoons, creaking of newspapers and books, rocking of chairs, squeaking of boots, sounds caused by articles thrown down from the hands or the lap, or by sudden jerking or starting from the seat ; and worse still is the sharp shriek caused in response to every trifling fright. All sudden startling noises must be avoided.

In walking, in conversation, in giving instructions to assistants, in moving about furniture (such as chairs, couches, screens, surgical instruments, plates, knives, forks, coals, and fire-irons), in opening and shutting doors, windows, and presses, no more noise should be made than is absolutely unavoidable.

Coals should never be broken in the sick-room, and they should be placed on the fire noiselessly. This is best done by wrapping in brown paper, each parcel being sufficient for once supplying the fire when required. Housemaids' gloves may also be used for moving about coals silently. Firewood or walking-sticks may be substituted for pokers. Fire-irons must be so placed as not to cause anyone to stumble over them. Ice should be broken by driving a nail, strong pin, or stout knife into it at the desired point.

All creaking of doors, handles, lockers, and windows must be prevented. Vaseline is often useful to lubricate hinges that are rough and creaky. Curtains and blinds must not flap. Vernon's patent noiseless china is a great boon in the sick-room. The india-rubber castors prevent not only all noise, but also that most harrowing sensation known as "creeping of the flesh," to which patients become subject in conditions of exhaustion. Many patients, especially of the male sex, will not tell you that they are subject to this peculiar feeling, because popularly it is associated with childishness ; but if you watch their expression, you will frequently find evidences of its existence in prolonged disease. All tables, especially such as have marble slabs, should be covered, and plates and other articles should be lifted clean off them—not pulled or pushed about on the grating surface. It is important to remember that a watch, when placed on a table or a chest of drawers, will have its tick intensified and conducted to the patient with painful distinctness. Sometimes a watch placed under the pillow gets so situated on one of the bars of an iron bedstead as to communicate its tick to the whole framework, and cause restlessness by thus ringing the bed.

In moving about in the sick-room, please walk with a free, silent step. Do not shuffle on the floor or stamp it with your heels. Remember this caution, especially in nursing male patients belonging to such classes as are hard-worked mentally. No blister irritates him more than this unnecessary disturbance made by the attendants. He will not complain on the point; but, nevertheless, the harm done to him is very great. Remember not to move about the room during the examination of any case by the medical attendant.

While enjoining strict silence in the sick-room, it must be understood that there must be no exaggerated desire shown to make the sick chamber into a deaf and dumb asylum. There is a fussy, sham quietness which disturbs the patient far more than the loudest noise. There should be no great affectation of kindness, sympathy, mysterious, sly, tiptoe movement, or blankness of look and expression, shown in the sick-room. These have all a funereal aspect, and will be so construed by the patient, who not only "hears his burial talked of by his friends," but sees it foretold by every expression and movement of theirs. There must be no whispering in the sick-room, or just within the hearing of the patient. If there is anything that must be said, speak it out, even if you must do it at the top of your voice. No conversation should be carried on in the sick-room where there is a serious case. No speaking should be allowed just within the hearing of the patient, as this is more injurious than a conversation carried on in the room itself. Do not allow anyone to speak to the patient from behind a half-opened door. If you can possibly help it, avoid all mysterious communication with the medical attendant. If anything likely to be disagreeable to the patient has been blurted out by mistake, it is best to smooth it over by a full explanation, given to all appearance in candour, and not to look profoundly wise in the attempt to conceal it. No nurse should indulge in the clumsy habit of thinking aloud.

It is important to remember that patients who are apparently unconscious—as in fits, coma, under chloroform, etc.—may be acutely sensitive to sounds, and will hear, remember, and quite sincerely misconstrue what has been whispered within their hearing.

All directions about changes of bedding, methods of rendering assistance to each other, and dressing of patients should be given to the nurses beyond the hearing of the patient. All fault-finding and reprimanding should also be done in silence and secrecy, certainly *never* within the hearing of male patients, as the wisest and most experienced of that irreverent sex declared more than once his readiness, under certain conditions, to dwell in the corner of the house-top in preference to a wide house.

Never run any chance of awakening a patient from his first sleep. It is the soundest and most refreshing portion of sleep. Early to bed and *late* to rise is the rule in disease. The more the sick sleep the greater is their capacity for sleep.

Patients who are really ill do not desire to be spoken to very much, but in this matter the racial and personal peculiarities of each case must be observed. Except such as make a luxury of illness, it is seldom that a patient desires to be made the subject of conversation. You should discourage the infliction of the numerous blisters on the patient in the way of "cheering up," "sympathy," and "advice" given at large. The flushing which follows such vigorous applications is, unfortunately, mistaken by the visitor as signs of their company having done good to the sick. One who is seriously ill appreciates loud manifestations of sympathy and fussy attentions just as much as an honest man in difficulties appreciates to be kindly reminded of his pauperism.

In your own conversation with a patient, remember not to startle him or obtrude any remarks while he is engaged. Do not speak to a weak patient while he is moving about or standing. Always sit down in full view of the patient, so that he can see you without resorting to any painful gymnastic manœuvres. If you can help it, show no evidence of any hurry while he is speaking to you. Do not lean upon, sit, or shake the bed of a weak patient or of one in pain, such as cases of rheumatism, gout, periostitis, joint diseases, etc. It is a general rule not to lean against anything in the sick-room, such as chairs, tables, the mantelpieces, etc.

The voice must be pitched according to circumstances. This is a point which requires constant attention on the part of the nurse. It was a touch of true genius, kept well under the control of facts, that made Lear, in his bodily and mental breakdown and utter helplessness, remember that Cordelia's

"voice was soft,
Gentle, and low."

There is no truer index of feeling in the sick-room than the human voice. The ring of it alone will tell you who the patient's real friends are. The same pitch, however, will be painful or pleasant to one and the same patient under different circumstances. In most diseases hearing becomes acute, sometimes painfully acute; but in the same patient it may be acute or dull at different hours of the day. Your eye, by watching the patient's expression when addressed, must guide you in this matter.

In certain conditions the patient must not be allowed to speak much or at all. Such are cases of acute diseases of the liver, peritoneum, bowels, and certain diseases of the larynx and chest. You should encourage such a one to make his wants known through signs or by writing.

Music is rarely of any value in the sick-room. In no case of serious disease can the nurse allow any instrument to be played upon. In some chronic cases continuous music may be allowed; but interrupted notes, like those of a piano, are almost never admissible.

A patient who is seriously ill will be benefited neither by reading nor by being read to. In head diseases, or in conditions where there is nervous exhaustion, absolute and persistent rest to all the brain functions must be insisted upon by you. No trained nurse would now-a-days allow a patient with joint disease, periostitis, etc., to use the limb; but few seem to realise that the same principle of nursing holds good for a damaged brain. All excitement in the way of talking, reading, thinking, and emotion is just as much injurious to a diseased brain as walking is to a damaged knee-joint. In all serious brain diseases, sudden or loud noises must be avoided. Long after the disease is supposed to be cured we may have relapses if the patient is subjected to startling noises. When reading may be indulged in by the patient without any risk of harm, he should be enabled to do so with perfect comfort. The light should be suitably arranged for the purpose.

If the patient is to be read to from a book, the process must be slow, distinct, of short duration, and a continuous one—that is, not merely in catches here and there. It is well to remember that certain productions, especially brilliant review articles, books of the season, or society novels of a few years back, have great virtues as sleeping-draughts. If read with the due degree of monotony, they are effectual soporifics. Few patients can stand long an ordinary dose of them. The sleep so produced is of the soundest and most refreshing kind that ever visits any mortal.

*(To be continued.)*

